# Dr. Roger Williams on Oöphorectomy for Cancer of the Breast

**Published:** 1902-01-18

**Authors:** 


### DR. ROGER WILLIAMS ON OÖPHORECTOMY FOR CANCER OF THE BREAST.

Dr. Roger Williams comments very severely on the proposal to remove the ovaries in the hope of modifying the course of cancer of the breast. He holds strongly to the point which he has made before, namely, that ovariotomy actually predisposes to the occurrence of carcinoma. He says that removal of the ovaries tends to favour, rather than prevent, the development of cancer; and therefore that "this craze for oöphorectomy is a horrible mistake."

